# Prevalence, Awareness, Treatment, and Control of Hypertension among Saudi Adult Population: A National Survey

**DOI:** 10.4061/2011/174135

**Published:** 2011-09-06

**Authors:** Abdalla A. Saeed, Nasser A. Al-Hamdan, Ahmed A. Bahnassy, Abdelshakour M. Abdalla, Mostafa A. F. Abbas, Lamiaa Z. Abuzaid

**Affiliations:** Department of Community Medicine, Faculty of Medicine, King Saud Bin Abdulaziz University for Health Sciences, King Fahad Medical City, P.O. Box 59046, Riyadh 11525, Saudi Arabia

## Abstract

This cross-sectional study aimed at estimating prevalence, awareness, treatment, control, and predictors of hypertension among Saudi adult population. Multistage stratified sampling was used to select 4758 adult participants. Three blood pressure measurements using an automatic sphygmomanometer, sociodemographics, and antihypertensive modalities were obtained. The overall prevalence of hypertension was 25.5%. Only 44.7% of hypertensives were aware, 71.8% of them received pharmacotherapy, and only 37.0% were controlled. Awareness was significantly associated with gender, age, geographical location, occupation, and comorbidity. Applying drug treatment was significantly more among older patients, but control was significantly higher among younger patients and patients with higher level of physical activity. Significant predictors of hypertension included male gender, urbanization, low education, low physical activity, obesity, diabetes, and hypercholesterolemia. In conclusion prevalence is high, but awareness, treatment, and control levels are low indicating a need to develop a national program for prevention, early detection, and control of hypertension.

## 1. Introduction

The importance of high blood pressure as a major cause of common serious diseases and deaths has been recognized in most countries particularly Western countries [1]. Hypertension affects more than a quarter of the global adult population including Kingdom of Saudi Arabia (KSA). It is projected in year 2025 to increase by 24% in developed countries and 80% in developing countries [2]. The increase is expected to be much higher than these projections [3]. National surveys of prevalence, awareness, treatment, and control provide basis for assessing the burden of hypertension in the community. These surveys showed that many hypertensives were unaware of their disease, many of the aware were not on treatment, and many of the treated are not controlled particularly in developing countries [4–6]. The increase in hypertension prevalence will invariably lead to dramatic rises in the incidence of cardiovascular diseases and their consequences, which has the potential to overwhelm health care systems [3]. It will also have financial implications for provincial drug plans because there is increasing evidence that the majority of patients with hypertension will require two or more drugs to achieve blood pressure control [7]. Hence it is vital to assess the burden of hypertension and associated risk factors as a prerequisite for meaningful prevention and control strategies. The present study aims at assessing the prevalence, level of awareness, treatment, control, and identification of risk factors and significant predictors of hypertension among Saudi adults.

## 2. Subjects and Methods

This is a cross-sectional community-based study covering the whole of KSA in 2005. The WHO STEPwise approach to Surveillance of Noncommunicable Disease (NCD) risk factors (STEPS) was the basis for conducting the survey and for collecting the data [8, 9].

## 3. Study Population

All Saudi population from all the 20 health regions of the country aged 15–64 years was included.

## 4. Sampling

A multistage stratified cluster random sampling technique was used to recruit the study subjects. Stratification was based on age (five groups with ten years interval), gender (2 groups male/female), and health regions of the country (20 regions). Based upon proposed methodology of the STEPS approach, a sample size of 196 was calculated for each of these ten strata. A list of all Primary Health Care Centers (PHCCs) in each region was prepared, and 10% of these PHCCs were randomly chosen and allocated regional sample proportionate to the size of their catchment population in sampled PHCCs. To identify the households, a map of the health center coverage area was used to choose the houses. Each house was assigned a number, and a simple random draw was made.

## 5. Data Collection

### 5.1. Tool Used

Data was collected using the STEPS approach which includes a questionnaire and physical measurements plus biochemical measurements covering hypertension and other chronic diseases and risk factors. The questionnaire was translated into Arabic by a team of physicians and was back translated to ensure the accuracy of translation. Arabic instrument was pretested on 51 eligible respondents for wording and understanding of the questions. Necessary adjustments and corrections were made in the instrument in light of the pretest before using it. The questionnaire included sociodemographic data and history of blood pressure and blood pressure measurements in addition to other chronic diseases and risk factors.

### 5.2. Data Collectors

Data was collected by 54 male and 54 female collectors who work in teams. Each field team was made up of four persons: a male data collector, a female data collector, a driver, and a female assistant. Data collection teams were supervised by a hierarchy of local supervisor, regional coordinators, and national coordinator.

### 5.3. Training of Data Collectors

All individuals involved in data collection attended a comprehensive training workshop that included interview techniques, data collection tools, practical applications, and field guidelines.

## 6. Blood Pressure Measurement

The measurements of blood pressure (BP) were taken using a digital sphygmomanometer. Before taking the measurements, the respondent was advised to sit quietly and rest for 5 min with the legs uncrossed and the right arm free of clothing. Then, the right arm was placed on the table with the palm facing upwards. The appropriate cuff size was selected. The artery position mark (ART) was aligned with the brachial artery. The cuff was wrapped snugly and fastened securely. The cuff was kept at the same level as the heart during measurement. Taking measurement involved the following steps: pushing the “START” button enabled automatic inflation of the cuff and display of the reading of systolic blood pressure and diastolic blood pressure readings, which were recorded. A second reading was taken after five minutes from the first, and a third reading was taken after five minutes from the second one.

## 7. Definition of Hypertension

The subject is labeled hypertensive if the average of the 3 BP measurements was 140 mmHg or above for systolic and/or 90 mmHg or above for diastolic blood pressure or the subject is a known hypertensive diagnosed previously by a health professional.

## 8. Data Management and Analysis

Questionnaires collected from the field were reviewed by team leaders assigned to each team before submitting them to the headquarters for data entry. Double entry of the questionnaires was performed using EPI-INFO 2000 software and EpiData software developed by the Menzies centre for validation. After data entry, data cleaning was conducted. New variables were defined by adopting the standard Steps variables (STEPS Data Management Manual, Draft version v1.5, October 2003). Data analysis was conducted using SPSS (Version 17) software. The number of participants' responses used in the discrete statistical analyses varied due to missing data for certain variables. Descriptive statistics and univariate and multivariate analysis were performed for associations and risk factors for hypertension prevalence, awareness, treatment, and control. Chi squared test was used to find if there is any association between each of hypertensive, aware hypertensive, on drug treatment, and controlled subjects with different demographic variables and other associated risk factors. Logistic regression model was used to predict hypertension from the associated risk factors. Odds ration and its 95% confidence intervals were done. Chi squared test and logistic regression were used as appropriate, and *P* value was set to <0.05 throughout the study.

## 9. Ethical Clearance and Confidentiality

The protocol and the instrument of the surveillance were approved by the Ministry of Health, Center of Biomedical Ethics and the concerned authorities in the Kingdom. Informed consent of all subjects was obtained. Confidentiality of data was assured and that data will be used only for the stated purpose of the survey. Further details of the method used and sampling procedures can be found in STEPS documents [8, 9].

## 10. Results

Of the total 4758 subjects who participated in the study, about 51% were females. A total of 1213 were hypertensives giving a prevalence of 25.5% (27.1% for males and 23.9% for females). Of all hypertensives, 542 (44.7%) were known patients confirmed by health professionals and 671 (55.3%) were unaware of their disease. They were newly detected during the survey. Of the aware hypertensives, 389 (71.8%) were under drug treatment and 144 (37.0%) of them were controlled (BP under 140/90 mmHg). Tables 1 and 2 show Chi squared test results of the prevalence, awareness, drug treatment, and control of hypertension among the study participants according to some demographic, life style, and biochemical variables. There is a significant association between hypertension and each of the following: gender, age, region, educational level, and participants' occupation (*P* < 0.001). The prevalence of hypertensive males was significantly higher than hypertensive females. As age increases, the prevalence of hypertension significantly increases. People in the central region of the KSA have higher prevalence than other regions. Uneducated participants showed higher prevalence than others. Retired participants showed the highest prevalence compared to other occupations. For smoked tobacco products, there is a significant association (*P* = 0.002) between hypertension and current smoking status where nonsmokers have significantly higher hypertension prevalence. Among current smokers themselves, nondaily smokers have lower hypertension prevalence compared to daily smokers, but differences were not significant. Further analysis showed no significant association between hypertension and smokeless tobacco product use currently or in the past. Subjects classified as having high physical activity level have significantly lower prevalence than others (*P* < 0.001). As body mass index increases, prevalence of hypertension increases (*P* < 0.0001). Diabetics and hypercholesteremic subjects have significantly higher hypertension prevalence (*P* < 0.001). Higher awareness of hypertension was significantly more among females, older subjects, residents of Eastern Region, housekeepers, and, with the presence of diabetes mellitus and an increasing level of physical activity. Applying drug treatment was significantly more among older patients, but control was significantly higher among younger patients and patients with higher level of physical activity.  Table 3 shows a logistic regression model to predict being hypertensive from some studied variables adjusting for age of the participants. The model shows that being in the central region of KSA, male, retired, diabetic, and having a high body mass index, high cholesterol are significant predictors for having hypertension.

## 11. Discussion

This study, to the best of our knowledge, is the first to present authentic nationwide current data about prevalence, awareness, treatment, and control of hypertension among adults in one report. It is hoped that findings can be of help in efforts to control the disease.

### 11.1. Prevalence, Risk Factors, and Predictors

This study found that hypertension affects more than a quarter of the adult population in agreement with a previous national study [4]. A similar pattern was reported from other developed and developing countries despite an average population age that is some 10–15 years lower than those of developed countries [1–7, 10–15]. This shows that the global burden of hypertension is considerable, and it appears to be increasing which is a cause for concern for health and other concerned authorities. This requires an urgent intervention plan. This study showed the significant relation of hypertension with advancing age in both sexes in agreement with national and international studies in almost all populations with diverse geographical, cultural, and socioeconomic characteristics [1–7, 10–15]. This indicates the degenerative aging process resulting in thickening and loss of elasticity of arteries which is a contributing factor for high blood pressure [16]. The tendency of males to have higher blood pressure in this study appears to be in agreement with some national and regional studies [4, 10, 11]. Overall worldwide prevalence of hypertension, however, showed no significant gender difference [2]. Reported gender differences may result from biological differences, but they may be due to other sociodemographic, comorbidity, obesity confounding effect. This study found that significant predictors of hypertension (Multiple Logistic Regression Analysis) included in addition to age and gender, obesity, urbanization, retirement from work, diabetes mellitus, and hypercholesteremia. Other significant risk factors included low educational and physical activity levels. Hypertension was significantly associated with lower educational level, retired and unskilled laborers in KSA and other Gulf and Asian countries [4, 11, 17–19]. These differences may be due to psychological or specific occupational hazards or due to some confounders associated with hypertension and both education and occupation [19]. Hypertension is significantly associated with lower levels of physical activity in agreement with several studies [20, 21]. Rapid developments in standards of living and increased mechanization and use of computer and telecommunication technology resulted in low levels of physical activity and sedentary living resulting in that most adults did not reach recommended physical activity targets [22]. This is a cause for concern as the health benefits of physical activity are well established in prevention and control of many NCDs including hypertension. Efforts are needed to encourage all sectors of the community to be physically active after preparing the necessary culture-sensitive facilities. In this context we fully support the strategies prepared by national experts to improve physical activity and lifestyle patterns in the community of KSA and other Arab countries [23]. Family income is not significantly associated with hypertension in this study. The previous survey in the country showed that hypertension was significantly more in the lowest-income group [4]. Studies in other communities reported that in both men and women, the income distributions of blood pressure and hypertension were nonlinear, indicating elevated levels in low- as well as in high-income groups [19]. Low and high incomes may be associated with psychological tensions which may be associated with hypertension. Education, occupation, and income are all related to socioeconomic status (SES). Low SES is associated with elevated rates of blood-pressure-related cardiovascular disease [24, 25]. The inaccuracies of self-reported data on income in some studies may also explain the inconsistent association of income with hypertension. Our findings showed significant geographical variation in hypertension prevalence where the central region had the highest and the southern region had the lowest prevalence of hypertension. The central region is highly urbanized, industrialized, and developed compared to the southern region. Geographical variations in the prevalence of hypertension were reported by many studies in different regions of the world [4, 11, 26–28]. These regional variations in blood pressure may also be related to variation in socioeconomic, demographic, and dietary in addition to the geographic characteristics. Studies showed that hypertension was threefold more in diabetics and significantly more in obese and those with high levels of total cholesterol, which is in agreement with our findings [29–32]. The situation is very worrying when we know that the prevalence of obesity, diabetes, and dyslipidemia among adults in KSA has reached alarming magnitudes affecting more than a quarter of the population [33, 34]. This study showed significant association with current smoked tobacco product use but not with smokeless or current daily tobacco use. Smoking status is not a predictor of hypertension in this study. Smoking has variable, inconsistent, and at times contradictory association with hypertension as reported by many studies. Population-based studies in some communities showed that hypertension was associated with smoking in a dose-response manner when characterized as number of years of smoking and lifetime cigarette consumption, but was not associated with current smoking status [35]. Others, however, have failed to provide any evidence or even demonstrate a negative association [36]. We think it is always wise to ask about smoking habits and encourage smokers to quit. Even if smoking is not definitely associated with hypertension, it is definitely associated with CVD and many other morbidities.

### 11.2. Awareness

A worrying finding in this study is that less than half of the hypertensives were aware of their disease at the time of the survey in agreement with a previous study [37]. In other countries a quarter to more than half of hypertensives were unaware of their disease and in some developing countries a devastating 85% unawareness rate was reported [38–45]. Higher awareness of hypertension in this study was significantly more among females, older subjects, residents of Eastern region, housekeepers, diabetics, and those with increasing level of physical activity. A previous study in the country found that independent predictors of a lack of awareness of hypertension were an age of at least 45 years, male gender, and obesity [37]. Females appear to be more concerned with their health compared to males, and this is expected since health status may affect body image which is more important for females. Older people, obese, and diabetics who are more at risk are also expected to be more concerned with their health status.

### 11.3. Treatment and Control

This study found that of all aware hypertensives about 72% were on drug treatment but only 37.0% of them were adequately controlled. These findings, although not satisfactory, are better than the findings of another study which found that of the 28.2% who were receiving treatment, only 3.7% had their blood pressure adequately controlled [37]. Control was achieved in 20–40% in many countries including some developed countries, which is in agreement with our findings [46]. This increase in awareness and control of BP is a welcome finding. Efforts are needed for further improvement by measures such as health education and screening services. Control in this study was significantly associated with younger age groups, secondary education, and physically more active hypertensives. No significant gender or regional differences were detected. Studies reported that control of hypertension was significantly higher among older than among younger individuals, among women rather than men, and among residents of urban rather than of rural areas, and it increases with educational level [41]. These may be expected findings since younger persons usually suffer less comorbidity which may facilitate hypertension control. Females may be more concerned with their health status as discussed before. Physical activity is a nonpharmacological treatment modality, and physically more active patients may also follow other positive lifestyle habits favoring hypertension control.

In summary this study highlighted a disturbing situation with hypertension affecting more than a quarter of the adult population, more than half of them were unaware of their disease, and less than 40% of those under drug treatment were controlled as judged by their blood pressure measurement of less than 140/90 mmHg. Despite the availability of a large number of effective and well-tolerated antihypertensive drugs and other modalities, blood pressure control rates are not satisfactory on a national and global scale [42]. The low level of awareness, treatment, and control is a global problem, but it is much serious in KSA and other developing countries necessitating urgent action. Efforts to combat the hypertension problem should be comprehensive including all sectors of the community. Special attention will be given to groups most affected and those least aware or treated. Such programs can include interventions to improve the knowledge, attitude, and behaviors of all sectors of the community including patients and health professionals in the context of prevention, early diagnosis, adherence to treatment, and control of hypertension. Such comprehensive and integrated program of interventions was effective in tackling the prevalence of hypertension and improved the awareness, treatment, and control rates of this disorder in a developing country setting [47].

### 11.4. Study Limitations

Comparisons of prevalence of hypertension among population groups in different countries are hindered by the different methodologies and definitions used. Information bias may have been present with respect to both recall of hypertension diagnosis and treatment and the validity of some of the predictor variables. We hope that the extent of the bias is minimal due to the confidentiality assured, proper training of all manpower involved in the survey, and the health objectives of the survey. Three measurements of BP were taken during one diurnal visit. Nocturnal variations and white-coat effect were not ascertained as no home monitoring of BP measurements was available.

## 12. Conclusion

The high prevalence of hypertension and the low prevalence of awareness, treatment, and control all are of high importance for all health professionals and politicians. There is a need for further research to document the impact of dealing with the modifiable risk factors and predictors of hypertension in reducing the burden of hypertension and its consequences. These results underscore the urgent need for developing a high blood pressure screening and education program to coordinate the effort of early detection, prevention, and treatment of hypertension in KSA.

## Figures and Tables

**Table 1:** Hypertension prevalence, awareness, treatment, and control according to sociodemographic characteristics of subjects*.

Variable (*n*)	Hypertensives *n* (%) 1213 (25.5)	Aware hypertensives *n* (%) 542 (44.7)	On drug treatment *n* (%) 389 (71.8)	Controlled *n* (%) 144 (37.0)
Gender:				
Male (2340)	634 (27.1)	236 (37.3)	175 (74.2)	56 (32.0)
Female (2418)	579 (23.9)	306 (53.3)	214 (69.9)	88 (41.1)
*P value	0.013	<0.001	0.662	0.208

Age (years)				
15–24 (1076)	95 (8.8)	19 (20.9)	5 (26.3)	3 (60.0)
25–34 (1130)	146 (12.9)	57 (12.9)	20 (35.1)	15 (75.0)
35–44 (1167)	304 (26.0)	117 (38.6)	73 (62.4)	35 (47.9)
45–54 (841)	355 (42.2)	185 (52.1)	147 (79.5)	56 (38.1)
55–64 (544)	313 (57.5)	164 (49.7)	144 (87.8)	35 (24.3)
*P value	<0.001	<0.001	0.662	0.021

Region				
Central (1139)	379 (33.3)	170 (45.3)	122 (71.8)	33 (20.1)
Eastern (706)	168 (23.8)	84 (50.0)	56 (66.7)	25 (38.5)
Northern (455)	128 (28.1)	48 (38.1)	36 (75.0)	12 (33.3)
Southern (1001)	207 (20.7)	76 (36.7)	54 (71.1)	23 (42.6)
Western (1457)	33 (22.7)	164 (49.7)	121 (73.8)	51 (42.1)
*P value	<0.001	0.012	0.279	0.379

Education				
None (1256)	482 (38.4)	253 (52.5)	204 (80.6)	68 (33.3)
Primary (1220)	310 (25.4)	141 (45.8)	110 (78.0)	40 (36.4)
Intermediate (754)	137 (18.2)	48 (35.3)	23 (47.9)	10 (43.4)
Secondary (791)	134 (16.9)	38 (28.8)	15 (39.5)	11 (73.3)
University (608)	127 (20.9)	49 (39.2)	25 (51.0)	10 (40.0)
Vocational (120)	19 (15.8)	13 (68.4)	12 (92.3)	5 (41.7)
*P value	<0.001	<0.001	0.055	0.002

Occupation				
Governmental (1371)	234 (23.6)	124 (38.4)	80 (64.5)	38 (47.5)
Nongovernmental (455)	143 (31.4)	54 (38.0)	43 (79.6)	12 (29.6)
Student (469)	55 (8.5)	8 (15.4)	1 (12.5)	0 (0)
Housekeeping (1759)	492 (28.0)	269 (54.9)	189 (70.3)	76 (39.2)
Retired (308)	157 (51.0)	73 (46.5)	66 (90.4)	17 (25.8)
Unemployed (210)	41 (19.5)	14 (34.1)	10 (71.4)	1 (10.0)
*P value	<0.001	<0.001	<0.01	0.244

Income (Saudi Riyals)				
<3000 (1492)	392 (26.3)	165 (42.2)	130 (78.8)	48 (39.9)
<7000 (1011)	240 (237)	104 (43.9)	75 (72.1)	27 (36.0)
<10000 (1329)	317 (23.9)	138 (43.5)	94 (68.1)	40 (42.6)
<15000 (443)	110 (24.8)	52 (47.3)	34 (65.4)	10 (29.4)
15000 + (229)	58 (25.3)	32 (56.1)	23 (71.9)	10 (43.5)
*P value	0.554	0.352	0.943	0.897

*P value using Chi squared test.

**Table 2:** Hypertension prevalence, awareness, treatment, and control according to some life style characteristics, diabetes, and cholesterol level*.

Subjects *n* (%) Variable (*n*)	Hypertensives *n* (%)	Aware hypertensives *n* (%)	On drug treatment *n* (%)	Controlled *n* (%)
Current smoking				
Yes (611)	127 (20.3)	48 (38.1)	24 (50.0)	13 (54.2)
No (4140)	1083 (26.2)	493 (45.7)	365 (70.0)	131 (35.9)
*P value	0.02	0.062	0.128	0.250

Currently daily smoker				
Yes (528)	107 (20.3)	39 (36.8)	19 (48.7)	8 (42.1)
No (73)	19 (26.0)	8 (42.1)	4 (50.0)	4 (100)
*P Value	0.163	0.432	0.969	0.288

Physical activity level				
High (764)	139 (18.2)	56 (40.3)	40 (71.4)	13 (32.5)
Moderate (771)	180 (23.3)	76 (42.5)	58 (76.3)	24 (41.4)
Low (826)	826 (26.9)	366 (44.5)	258 (70.5)	103 (39.9)
*P Value	<0.001	0.607	0.918	<0.001

Diabetes mellitus				
Yes (712)	361 (53.6)	228 (59.8)	175 (70.8)	47 (26.9)
No (3945 )	809 (20.5)	307 (38.1)	215 (68.4)	94 (44.8)
*P value	<0.001	<0.001	0.392	0.017

BMI				
Normal	19 (16.0)	72 (37.9)	52 (72.2)	23 (44.2)
Grade 1	385 (26.1)	160 (41.9)	129 (80.6)	44 (34.1)
Grade 2	496 (35.0)	241 (48.8)	163 (67.4)	60 (38.8)
Grade 3	100 (41.8)	56 (57.1)	43 (76.8)	16 (37.2)
*P value	<0.001	<0.001	0.705	0.867

Cholesterol				
Elevated	316 (36.5)	147 (46 > 7)	112 (76.2)	40 (35.7)
Normal	846 (23.3)	374 (44.3)	270 (72.2)	100 (37.0)
*P value	<0.001	0.258	0.717	0.868

*P value using Chi squared test.

**Table 3:** Logistic regression model to predict hypertension from some studied variables adjusting for age.

Variable	B	S.E of *β*	*P* value	Odds ratio	95% C.I. Odds ratio
Region					
Central	0.437	0.105	<0.0001	1.55	1.27–1.89
Eastern	0.029	0.120	0.89	1.03	0.81–1.30
Northern	0.230	0.141	0.197	1.26	0.96–1.65
Southern	0.133	0.111	0.237	0.88	0.71–1.10

Gender (male)	−0.27	0.146	<0.0001	0.52	0.39–0.69

Current smoking Tobacco	0.203	0.10	0.051	1.23	0.99–1.50

Occupation					
Governmental	0.27	0.203	0.19	0.77	0.52–1.14
Nongovernmental	0.08	0.223	0.73	1.08 1.36	0.69–1.68 0.87–2.11
Housekeeping	0.03	0.23	0.12	1.88	1.19–2.94
Retired	0.63	0.23	0.006		

BMI	0.44	0.04	<0.0001	1.55	1.43–1.68

Diabetes mellitus	−1.103	0.09	<0.0001	3.01	2.51–3.61

Total cholesterol	0.165	0.3	<0.0001	1.18	1.11–1.25

Constant	0.41		0.71	0.57	
